# An empirical study of continuous participation intention in Chinese CrossFit participants: mediating roles of community belonging and sport commitment

**DOI:** 10.3389/fpsyg.2025.1674562

**Published:** 2025-09-26

**Authors:** Xuan Ji, Qianjin Wu

**Affiliations:** ^1^School of Physical Education, Shandong University, Jinan, China; ^2^Physical Education College, Shandong University of Finance and Economics, Jinan, Shandong, China

**Keywords:** CrossFit, exercise motivation, sense of community belonging, sport commitment, continuous participation intention

## Abstract

**Background:**

Most existing CrossFit®-related studies focus on populations in Europe and North America, with limited attention to Chinese participants. In China, the sport’s promotion faces barriers such as high intensity and cost, highlighting the need to identify key factors influencing sustained participation.

**Purpose:**

This study investigates the influence of intrinsic exercise motivation on the intention for continuous participation among Chinese CrossFit participants. Specifically, it examines the mediating roles of sense of community belonging and sport commitment. Additionally, the study contributes Chinese samples to CrossFit research and provides theoretical and practical insights to help CrossFit facilities in China enhance operations and improve member retention.

**Methods:**

A total of 568 Chinese CrossFit participants were recruited through online CrossFit communities using a random sampling approach. A validated and reliable questionnaire was developed to assess exercise motivation, sense of community belonging, sport commitment, and continuous participation intention. Data analysis was conducted using SPSS and AMOS.

**Results and conclusions:**

Intrinsic exercise motivation positively influenced continuous participation intention (*p* < 0.001), sense of community belonging (*p* < 0.001), and sport commitment (*p* < 0.001). Sport commitment also positively affected continuous participation intention (*p* < 0.001) and mediated the relationship between motivation and participation. However, the sense of community belonging (*p* = 0.156) neither significantly influenced participation intention nor mediated the relationship between the two variables. These findings highlight the critical role of sport commitment while suggesting limited mediating effects of community belonging among Chinese CrossFit participants. Theoretically, this study develops an integrated model linking intrinsic motivation, mediating mechanisms (community belonging and sport commitment), and continuous participation intention. By incorporating belongingness theory and sport commitment theory into the CrossFit context, it extends existing motivational frameworks to a non-Western population and enriches cross-cultural perspectives on exercise adherence.

## 1 Introduction

CrossFit, a registered trademark of CrossFit, Inc., is a form of high-intensity functional training (HIFT) developed by Greg Glassman. It was initially designed for military and tactical training and has since gained widespread popularity among the general public. Its core objective is to develop ten fundamental physical attributes—cardiovascular endurance, stamina, strength, flexibility, power, speed, coordination, agility, balance, and accuracy—through constantly varied, high-intensity functional movements (Glassman, 2002). CrossFit training is delivered through daily varied routines known as the “Workout of the Day” (WOD), which incorporate elements such as gymnastics, Olympic weightlifting, and cardiovascular exercises (Fisker et al., 2017). The scalability of load intensity according to individual fitness levels has contributed to its widespread accessibility. Since the founding of CrossFit Inc. in 2000, the sport has grown into a global fitness phenomenon, expanding to 142 countries with over 14,000 affiliated gyms (Dominski et al., 2020).

Accompanying CrossFit’s global expansion, academic interest in the sport has grown. In the field of physiological and health risk research, Adhikari et al. and Meyer et al. reported a strong association between CrossFit training and the incidence of rhabdomyolysis (Adhikari et al., 2021; Meyer et al., 2021). Furthermore, several studies have identified a high incidence of upper extremity injuries related to CrossFit, particularly affecting the back, shoulders, and wrists (Hopkins et al., 2019; Tawfik et al., 2021; Nicolay et al., 2022). Notably, individuals with greater CrossFit experience and longer weekly training durations show a significantly increased risk of injury (Alekseyev et al., 2020). However, these studies do not address a central paradox: despite the elevated risk of injury, many participants continue to engage in CrossFit in the long term. This phenomenon of “risk coexisting with persistence” underscores the limitations of current research in examining the motivations and mechanisms sustaining CrossFit participation.

A limited number of studies on psychosocial aspects have provided valuable insights, but they remain constrained by significant limitations. For instance, Dawson (2017) argues that CrossFit demands substantial physical and mental commitment, characterizing it as “greedy.” Bailey et al. (2019) applied Schein’s organizational culture framework to analyze CrossFit’s culture, finding that, although participants’ personal goals vary considerably, a shared objective is to enhance overall health and well-being. Current evidence suggests that CrossFit fosters a community environment where a sense of belonging and social connectedness are crucial for promoting behavioral change. Furthermore, the inherently social nature of CrossFit training helps meet individuals’ basic psychological needs (Fisher et al., 2017; Bycura et al., 2017; Feito et al., 2018). Whiteman-Sandland et al. (2018) found that CrossFit gym members reported significantly higher levels of community belonging than those in traditional gyms. They also emphasized the need for further research to assess how this sense of belonging influences members’ adherence to exercise. However, current studies have not clarified whether the sense of community and belonging in CrossFit culture affects enthusiasts’ continued participation in training.

Overall, although existing CrossFit research addresses musculoskeletal injury risks, lifestyle impacts, and psychosocial behaviors (Claudino et al., 2018), two significant limitations persist: First, the mechanisms driving the “intention for sustained participation” remain underexplored, particularly due to the lack of a comprehensive analysis linking “motivation → mediating variables → sustained participation.” Second, the role of community belongingness has not been sufficiently validated. Although current evidence highlights its importance in CrossFit, no empirical study has confirmed whether this factor mediates the relationship between “participation behavior” and the “intention for sustained participation” (Claudino et al., 2018; Heinrich et al., 2017; Whiteman-Sandland et al., 2018).

Additionally, existing CrossFit research reveals a notable regional imbalance. Most empirical evidence has been derived from Europe and North America, while studies in the Chinese context remain scarce. This gap is particularly notable, considering the challenges in promoting and developing CrossFit in China, which underscores the importance of localized investigations. CrossFit officially entered China in 2013 with the opening of its first authorized gym in Shanghai. Over the next decade, the number of authorized gyms peaked at nearly 200, before entering a period of decline. As of 2025, fewer than 80 authorized gyms remain in operation (including Taiwan). A significant factor contributing to this decline is that operating revenues cannot cover costs, as course fees constitute the primary income source for CrossFit gyms (commonly referred to as “boxes”). This trend reflects both the loss of CrossFit enthusiasts and a low sustained participation rate in China.

However, no existing research has examined the key factors leading Chinese participants to discontinue CrossFit, nor has there been a localized investigation into how long-term participation intentions can be sustained. This disparity between practical needs and academic inquiry underscores the theoretical significance and practical value of studying continuous participation intentions among Chinese CrossFit practitioners.

Building on these research gaps, the present study focuses on the continuous participation intentions of Chinese CrossFit participants, with two primary objectives. First, it examines the direct effect of exercise motivation on continuous participation intentions, thereby identifying the core motivational factors driving long-term persistence. Second, it introduces community belongingness and exercise commitment as mediating variables, clarifies their mechanisms within the pathway from exercise motivation to continuous participation intentions, and compares their relative influence in determining sustained participation. Beyond addressing these empirical questions, this study makes a theoretical contribution by constructing a comprehensive “motivation–mediator–outcome” model in the context of CrossFit, incorporating both belongingness theory and sport commitment theory. Furthermore, it expands sports participation research to a Chinese sample, offering cross-cultural evidence that complements the predominantly Western literature.

## 2 Literature review and hypothesis

Exercise motivation (EM) serves as a primary psychological driver of individuals’ engagement in physical activity, significantly influencing both the initiation and long-term maintenance of exercise behavior (Hu et al., 2025). According to Self-Determination Theory, motivation can be classified as either extrinsic or intrinsic. While external rewards and pressures shape extrinsic motivation, intrinsic motivation stems from personal goals, such as enhancing health, enjoying the activity, or fostering a positive self-image (Ryan and Deci, 2000). Existing literature indicates that exercise motivation is predominantly intrinsic and comprises five core dimensions: health motivation (HM), competence motivation (CM), fun motivation (FM), appearance motivation (AM), and social motivation (SM) (Chen et al., 2013; Liu et al., 2023). Empirical studies have demonstrated that higher levels of intrinsic motivation are positively associated with individuals’ intentions to maintain regular participation in physical activity (Teixeira et al., 2012; Wendling et al., 2018; Choi, 2023). Within the context of CrossFit, which emphasizes functional improvement over appearance, intrinsic factors such as fun, challenge, and health have been identified as key drivers of participation and adherence (Fisher et al., 2017). Therefore, we propose the following hypothesis:

*H1*: Exercise motivation positively influences the continuous participation intention of CrossFit participants.

A sense of community belonging (CB) represents a fundamental psychological need, encompassing the desire to feel recognized and valued within a social environment (Cao and Lyu, 2024). When individuals perceive a strong sense of community belongingness, they are more likely to adopt adaptive health behaviors (Dowd et al., 2014). Due to the technical complexity of CrossFit, training is commonly delivered through structured group classes led by coaches. This group-class training model fosters a community-centered culture that has become a hallmark of the CrossFit experience (Lautner et al., 2021; CrossFit, 2025). Prior research has shown that a strong sense of belonging enhances the sustainability of sports participation (Cao and Lyu, 2024). Thus, individuals with higher levels of exercise motivation may be more inclined to seek and develop a sense of community within CrossFit, which in turn can strengthen their long-term adherence to exercise. Based on this rationale, the following hypotheses are proposed:

*H2*: Exercise motivation positively affects the sense of community belonging among CrossFit participants.

*H3*: Sense of community belonging positively influences the continuous participation intention of CrossFit participants.

*H4*: Sense of community belonging mediates the relationship between exercise motivation and continuous participation intention.

Sport commitment (SC) refers to a psychological state that reflects an individual’s desire and determination to continue engaging in physical activity over time, emphasizing persistence and emotional attachment (Scanlan et al., 1993). Numerous studies have confirmed that exercise motivation positively influences sport commitment, and that individuals with greater commitment are more likely to sustain participation (Zahariadis et al., 2006; Garcia-Mas et al., 2010; Jeng et al., 2020; Choi, 2023). Given CrossFit’s high physical and technical demands, long-term involvement requires substantial personal investment and a shift in mindset from “I want to do it” to “I will keep doing it.” Therefore, sport commitment may serve as a key psychological mechanism linking exercise motivation to behavioral persistence. Accordingly, the following hypotheses are proposed:

*H5*: Exercise motivation positively influences sport commitment among CrossFit participants.

*H6*: Sport commitment positively affects continuous participation intention among CrossFit participants.

*H7*: Sport commitment mediates the relationship between exercise motivation and continuous participation intention.

**Figure 1:**
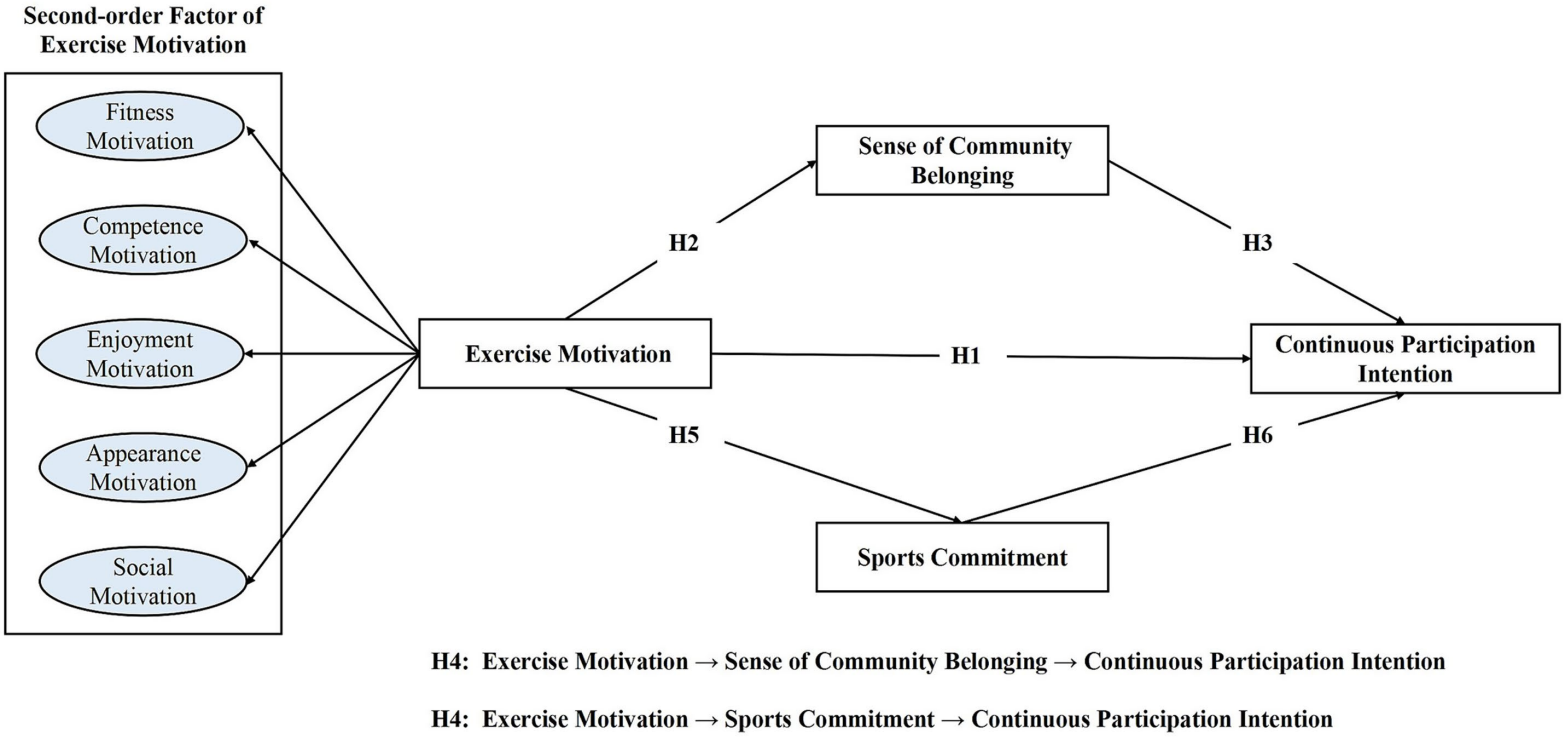
Conceptual model proposed in this study.

Based on the literature review and proposed hypotheses, a conceptual model was developed, as illustrated in Figure 1. The model integrates prior research findings, clarifies hypothesized relationships, and extends the theoretical framework to enhance understanding of the research topic.

## 3 Methodology

### 3.1 Measurement

To evaluate key constructs in this study, four validated measurement instruments were employed. All scales were administered in simplified Chinese and utilized a 7-point Likert scale ranging from 1 (strongly disagree) to 7 (strongly agree).

#### 3.1.1 Exercise motivation scale

Exercise motivation was assessed using the simplified Chinese version of the Motivation for Physical Activity Measure–Revised (MPAM-R), initially developed by Ryan et al. (1997) and later translated and adapted by Chen et al. (2013). The scale consists of 15 items covering five intrinsic motivation dimensions: health, competence, Fun, appearance, and social motivation (see Supplementary Table S1).

#### 3.1.2 Sense of community belonging scale

Sense of community belonging was measured using an adapted version of the General Belongingness Scale developed by Malone et al. (2012). Following the approach of Whiteman-Sandland et al. (2018), modifications were made to align with the study’s objectives and Chinese language conventions. The final scale included four items (see Supplementary Table S2).

#### 3.1.3 Sport commitment scale

Sport commitment was measured using the Chinese version of the Exercise Commitment Scale, which was translated and revised by Qiu et al. (2012) based on Wilson’s Exercise Commitment Scale (Wilson et al., 2000). Items were adjusted to reflect the specific context of Chinese CrossFit participants. The resulting scale contained five items (see Supplementary Table S3).

#### 3.1.4 Continuous participation intention scale

Continuous participation intention was assessed using a 3-item scale adapted from Choi (2023). Items were modified to suit the purpose of this study and the linguistic context, capturing participants’ intention to continue engaging in CrossFit over time (see Supplementary Table S4).

### 3.2 Data collection and sample description

This study employed an online questionnaire for data collection, conducted between February and April 2025. The questionnaire was distributed through the online communities of officially affiliated CrossFit® gyms across China. The research team first contacted gym managers and certified coaches, who then assisted in sharing the survey link with members. The link was primarily disseminated via WeChat groups and internal member platforms used for daily training communication. In addition, some coaches introduced the study during training sessions and provided a QR code for voluntary participation. All participation was voluntary and anonymous, with no material incentives offered.

To ensure that all respondents were actively training in CrossFit Inc.–affiliated environments, several screening items were included at the beginning of the questionnaire. Participants were asked to confirm whether they trained in an affiliated gym, whether their primary coach held a Level 1 or higher CrossFit® certification, and to provide the name of their training facility. Only respondents meeting all these criteria were retained in the final analytic sample.

A total of 568 questionnaires were distributed. Exclusion criteria were as follows: (1) completion time shorter than 2 min; (2) responses showing obvious patterns, such as selecting the same option throughout; and (3) failure to meet screening requirements, such as training in a non-affiliated gym or under an uncertified coach. After excluding 43 invalid or ineligible responses, 525 valid questionnaires remained, yielding an effective response rate of 92.4%. The demographic characteristics of the final sample are summarized in Table 1.

**Table 1:** Demographic characteristics of respondents.

Category		Number of participants	Percentage (%)
Gender	Male	258	49.1
	Female	267	50.9
Age	Under 18 years old	2	0.4
	18–25 years old	80	15.2
	26–35 years old	224	42.7
	36–45 years old	133	25.3
	46–55 years old	70	13.3
	Over 55 years old	16	3.0
Occupation	Civil Servants	63	12.0
	Private Enterprise Employees	182	34.7
	Freelancers	111	21.1
	Self-employed Persons	88	16.8
	Students	45	8.6
	Others	36	6.9
Monthly income (CNY)	Under 1,500	32	6.1
	1,500–3,000 (incl. 3,000)	29	5.5
	3,000–6,000 (incl. 6,000)	69	13.1
	6,000–10,000 (incl. 10,000)	222	42.3
	Over 10,000	173	33.0
Years of CrossFit® experience	Less than 1 year	117	22.3
	1–2 years (incl. 2 years)	105	20.0
	2–3 years (incl. 3 years)	136	25.9
	3–4 years (incl. 4 years)	75	14.3
	More than 4 years	92	17.5
Weekly frequency of participation	0–1 time	125	23.8
	2–3 times	234	44.6
	4–5 times	144	27.4
	More than 6 times	22	4.2

## 4 Results

### 4.1 Reliability and validity

Data analysis was performed using SPSS 26.0, and the reliability and validity results are summarized in Table 2. All scales demonstrated strong internal consistency, with Cronbach’s *α* coefficients exceeding 0.80. Composite reliability (*CR*) values ranged from 0.793 to 0.898, all of which exceeded the 0.70 threshold, confirming high internal reliability. Standardized factor loadings ranged from 0.591 to 0.858, and the average variance extracted (*AVE*) ranged from 0.566 to 0.707, both surpassing the 0.50 cutoff, thus indicating acceptable convergent validity.

**Table 2:** Measurement indicators, reliability, and validity of constructs.

Item		Factor loading	CR	AVE	Cronbach’s α
Exercise motivation	SM		0.878	0.706	0.918
	PM15	0.796			
	PM5	0.815			
	PM10	0.835			
	AM		0.843	0.641	
	PM12	0.697			
	PM2	0.711			
	PM7	0.884			
	CM		0.793	0.566	
	PM14	0.786			
	PM4	0.811			
	PM9	0.805			
	HM		0.811	0.591	
	PM1	0.808			
	PM11	0.855			
	PM6	0.856			
	FM		0.856	0.665	
	PM3	0.591			
	PM13	0.796			
	PM8	0.845			
Sense of community belonging	CB1	0.812	0.897	0.685	0.897
	CB2	0.835			
	CB3	0.837			
	CB4	0.827			
Sport commitment	SC2	0.851	0.898	0.688	0.901
	SC3	0.834			
	SC4	0.813			
	SC5	0.819			
Continuous participation intention	CPI1	0.858	0.879	0.707	0.908
	CPI2	0.825			
	CPI3	0.839			

SM, Social motivation; AM, Appearance motivation; CM, Competence motivation; HM, Health motivation; FM, Fun motivation; CR, Composite reliability; AVE, Average variance extracted.

Confirmatory factor analysis (*CFA*) was conducted using AMOS to assess the structural validity of the measurement model. As shown in Table 3, all model fit indices satisfied recommended standards: *χ^2^/df* = 2.009, *CFI* = 0.967, *TLI* = 0.963, *RMSEA* = 0.044, and *SRMR* = 0.0414, indicating a good overall model fit.

**Table 3:** Model fit indices of the measurement model.

Fit index	Recommended threshold	Observed value
Chi-square/df (*χ*^2^/df)	1 < χ^2^/df < 3	2.009
Comparative Fit Index (CFI)	>0.9	0.967
Tucker–Lewis Index (TLI)	>0.9	0.963
Root Mean Square Error of Approximation (RMSEA)	<0.08	0.044
Standardized Root Mean Square Residual (SRMR)	<0.05	0.0414

### 4.2 Direct effects in the second-order structural model

The standardized path coefficients of the structural equation model are presented in Table 4. Exercise motivation had a significant positive effect on continuous participation intention (*β* = 0.593, *p* < 0.001), supporting Hypothesis H1. It also significantly influenced sense of community belonging (*β* = 0.744, *p* < 0.001), indicating that individuals with stronger motivation are more likely to develop a stronger sense of community, thus supporting Hypothesis H2. However, sense of community belonging had no significant effect on continuous participation intention (*β* = 0.076, *p* = 0.156), suggesting that it does not directly influence sustained CrossFit engagement. Therefore, Hypothesis H3 was not supported. Exercise motivation also had a significant positive impact on sport commitment (*β* = 0.778, *p* < 0.001), supporting Hypothesis H5. Furthermore, sport commitment significantly predicted continuous participation intention (*β* = 0.245, *p* < 0.001), providing support for Hypothesis H6.

**Table 4:** Standardized path coefficients for the second-order structural model of exercise motivation among Chinese CrossFit participants.

Hypothesis	Path	Unstandardized coefficient	Standardized coefficient (β)	SE	CR	*p*-value
H1	EM → CPI	1.042	0.593	0.152	6.87	<0.001
H2	EM → CB	1.261	0.744	0.117	10.815	<0.001
H3	CB → CPI	0.079	0.076	0.056	1.42	0.156
H5	EM → SC	1.314	0.778	0.119	11.001	<0.001
H6	SC → CPI	0.255	0.245	0.061	4.173	<0.001

Significant results at *p* < 0.05 are highlighted. EM, Exercise motivation; CPI, Continuous participation intention; CB, Sense of community belonging; SC, Sport commitment; SE, Standard error; CR, Critical ratio.

### 4.3 Mediation analysis in the second-order structural model

Using Amos 26.0, model analysis was conducted with sense of community belonging and sport commitment as mediating variables. The second-order mediation paths of the exercise motivation model for CrossFit participants are presented in Table 5. The results of the standardized mediation analysis are summarized as follows: When sense of community belonging served as the mediator, the indirect effect of exercise motivation on continuous participation intention was not significant. The 95% confidence interval (CI = [−0.094, 0.169]) included zero, indicating no mediation. Therefore, Hypothesis H4 was not supported. In contrast, sport commitment significantly mediated the relationship between exercise motivation and continuous participation intention (CI = [0.139, 0.529]), as zero was not included in the interval. As exercise motivation also had a significant direct effect on continuous participation intention (see Table 4), sport commitment partially mediated this effect. Therefore, Hypothesis H7 was supported.

**Table 5:** Mediation effect results of the second-order structural model.

Hypothesis	Mediation path	Estimate	SE	95% CI (bias-corrected)	*p*-value	95%CI (percentile)	*p*-value
H4	EM → CB → CPI	0.100	0.087	[−0.089, 0.270]	0.259	[−0.094, 0.269]	0.269
H7	EM → SC → CPI	0.335	0.103	[0.140, 0.530]	0.002	[0.139, 0.529]	0.002

Mediation significance is determined by whether zero is excluded from the confidence interval. EM, Exercise motivation; CB, Sense of community belonging; SC, Sport commitment; CPI, Continuous participation intention; CI, Confidence interval; SE, Standard error.

### 4.4 Path analysis of the first-order motivation model

Based on the second-order factor model, exercise motivation—serving as an antecedent variable—positively influences the sense of community belonging, sport commitment, and continuous participation intention. To further investigate the specific dimensions underlying exercise motivation among CrossFit participants, this study develops a first-order factor model comprising five key dimensions: health, competence, fun, appearance, and social interaction. This model is intended to provide a clearer theoretical foundation for future research on CrossFit-related behavioral mechanisms and motivational structures.

Based on the results presented in Table 6, several conclusions can be drawn regarding the effects of five first-order motivation dimensions. With respect to the sense of community belonging, health motivation (*β* = 0.340, *p* < 0.001), competence motivation (*β* = 0.311, *p* < 0.001), and social motivation (*β* = 0.325, *p* < 0.001) showed significant positive effects. Fun motivation also had a significant effect, albeit weaker (*β* = 0.145, *p* = 0.002), while appearance motivation was not significant (*β* = 0.027, *p* = 0.546). For sport commitment (SC), health motivation (β = 0.368), competence motivation (β = 0.384), and fun motivation (*β* = 0.246) had significant positive impacts (all *p* < 0.001), whereas appearance motivation (*β* = 0.085, *p* = 0.060) and social motivation (β = 0.120, *p* = 0.007) did not exhibit consistently significant effects. Regarding continuous participation intention (CPI), all five motivational dimensions had statistically significant positive effects. Health motivation had the most decisive (*β* = 0.444), followed by fun (*β* = 0.314), competence (*β* = 0.298), and appearance motivation (*β* = 0.197), all with *p*-values < 0.001. Social motivation contributed the weakest effect (*β* = 0.125, *p* = 0.004), though still statistically significant.

**Table 6:** Path analysis results of the first-order exercise motivation model.

Path	Unstandardized coefficient	Standardized coefficient (*β*)	SE	CR	*p*-value
HM → CB	0.282	0.34	0.039	7.324	<0.001
CM → CB	0.263	0.311	0.040	6.578	<0.001
FM → CB	0.164	0.145	0.052	3.134	0.002
AM → CB	0.026	0.027	0.043	0.604	0.546
SM → CB	0.274	0.325	0.039	6.943	<0.001
HM → SC	0.295	0.368	0.038	7.780	<0.001
CM → SC	0.278	0.384	0.040	7.233	<0.001
FM → SC	0.272	0.246	0.054	5.051	<0.001
AM → SC	0.080	0.085	0.043	1.883	0.060
SM → SC	0.098	0.120	0.037	2.689	0.007
HM → CPI	0.351	0.444	0.038	9.336	<0.001
CM → CPI	0.242	0.298	0.038	6.443	<0.001
FM → CPI	0.343	0.314	0.055	6.267	<0.001
AM → CPI	0.184	0.197	0.043	4.323	<0.001
SM → CPI	0.101	0.125	0.036	2.853	0.004

HM, Health motivation; CM, Competence motivation; FM, Fun motivation; AM, Appearance motivation; SM, Social motivation; CB, Sense of community belonging; SC, Sport commitment; CPI, Continuous participation intention; SE, Standard error; CR, Critical ratio.

## 5 Discussion

This study aimed to investigate the influence of exercise motivation on the intention to continue participating in CrossFit among Chinese participants. The findings indicate that exercise motivation has not only a direct effect on continuous participation intention but also an indirect effect through sport commitment. However, the sense of community belonging neither directly influenced continuous participation intention nor served as a mediator. These results provide new insights into the motivational mechanisms underlying sustained exercise behaviors within the CrossFit context in China.

### 5.1 The effect of exercise motivation on continuous participation intention

Hypothesis H1 was supported, demonstrating that intrinsic motivation is positively associated with continuous participation intention. This finding aligns with prior findings from Western samples (Fisher et al., 2017) and adds empirical evidence from a Chinese population. Among the five motivational dimensions, health motivation exerted the most decisive influence, consistent with the conclusions of Laynes et al. (2022). This result may be interpreted in light of post-pandemic societal changes: recent global health crises have reshaped public health perceptions in China, leading to heightened awareness of immunity, disease prevention, and physical resilience. An empirical study of Chinese urban residents also found that health concerns significantly stimulate the desire to engage in regular physical activity (Zou et al., 2022). Given that CrossFit emphasizes functional movement and physical preparedness, its health-centered orientation closely aligns with public demand, making health motivation a primary driver of long-term engagement.

### 5.2 The mediating role of community belongingness

Hypothesis H2 was also supported, indicating that intrinsic motivation has a significant influence on the sense of community belonging. Notably, health motivation, competence motivation, and social motivation had more substantial effects, while fun motivation was weaker, and appearance motivation showed no significant impact. In contrast, Hypotheses H3 and H4 were not supported. Sense of community belonging neither directly affected continuous participation intention nor mediated the relationship between motivation and intention. This finding contrasts with previous studies emphasizing the role of belonging in promoting exercise adherence (Whiteman-Sandland et al., 2018; Cao and Lyu, 2024), suggesting that the dynamics of community influence in the Chinese CrossFit context may differ from those in other cultural settings.

This discrepancy may be attributed to three key factors. The first factor is economic constraints. While many Chinese CrossFit participants are high-income earners—with 75% earning over 6,000 yuan per month and 30% earning 10,000 yuan—this group (primarily aged 26–45) also faces financial pressures related to housing, childcare, and eldercare. Moreover, participation in CrossFit costs several times more than membership in traditional gyms. Therefore, despite a strong sense of community, the high training costs remain a significant barrier, hindering the conversion of “emotional identification” into “behavioral persistence.” Although no conclusive evidence currently demonstrates that economic pressures influence Chinese citizens’ participation in CrossFit, research from China indicates that time costs and financial expenditures for sports participation are significant determinants of overall physical activity engagement (Xu et al., 2022). In terms of its characteristics, CrossFit remains a niche sport in China with a relatively high technical threshold. Consequently, enthusiasts must undergo long-term training before receiving positive feedback on their skill improvement. However, existing research indicates that CrossFit training demands a strong sense of belief and identification (Dominski et al., 2020). Thus, this “delayed gratification” characteristic partly undermines practitioners’ confidence and weakens the immediate motivational effect of belongingness on sustained participation. Additionally, the relatively high injury risk associated with CrossFit (Nicolay et al., 2022; Lastra-Rodríguez et al., 2023) could further undermine the positive effects of community on participation intentions. Third, priority of needs. For many Chinese participants, tangible outcomes such as health improvement and skill acquisition are prioritized over emotional factors. In this context, a sense of community belonging is perceived as secondary or supplementary to other forms of identity. Even in highly interactive communities, individuals may discontinue participation if their core goals are not met.

### 5.3 The mediating role of exercise commitment

The results of this study support Hypothesis H5, confirming that intrinsic motivation has a significant influence on sport commitment. However, the strength of this relationship varies across motivational dimensions. Health motivation, competence motivation, and fun motivation all had significant positive effects on sport commitment, whereas appearance motivation and social motivation were not significant predictors. Specifically, health motivation operates through a logical progression: improvements in physical function align with personal health goals, thereby enhancing psychological commitment to continued participation. When participants experience tangible outcomes such as improved physical fitness or disease prevention, their engagement becomes internally reinforced.

Competence motivation contributes by fostering a sense of skill development and self-worth. The functional nature of CrossFit allows individuals to witness measurable progress—such as improved movement proficiency or achieving personal records—thereby deepening their recognition of the sport’s value. Although fun motivation plays a comparatively weaker role, it still contributes to sport commitment when combined with periodic achievements (e.g., mastering new movements) and positive social reinforcement (e.g., encouragement from coaches). These elements collectively reduce dropout tendencies and sustain long-term involvement.

In contrast, social motivation exhibited a non-significant effect on sport commitment, which may be attributed to the instrumental nature of interpersonal interactions in Chinese CrossFit communities. Communication among participants tends to center on technical aspects (e.g., training plans and techniques), with limited emotional bonding. As a result, social engagement does not translate easily into psychological commitment. Similarly, the limited influence of appearance motivation aligns with CrossFit’s emphasis on functionality over esthetics. While training enhances strength and endurance, these outcomes are loosely associated with mainstream body ideals, making appearance a secondary driver of sustained commitment.

Hypotheses H6 and H7 were also supported, indicating that sport commitment significantly influences continuous participation intention and mediates the relationship between intrinsic motivation and participation. This finding is consistent with Choi’s (2023) framework on the motivation–commitment–participation linkage but provides new empirical insights rooted in the Chinese CrossFit context. Sport commitment serves as a psychological anchor, anchoring behavioral consistency even in the absence of strong external factors such as a sense of community belonging. This finding reinforces the concept of “introverted commitment”—that is, commitment grounded in an individual’s personal bond with the sport rather than their social ties to the community.

This internally anchored commitment appears robust against variations in external social influences. For instance, CrossFit participants may relocate due to life or work changes but continue training in new venues if their commitment to the sport remains intact. Conversely, when commitment diminishes—such as when health goals are achieved or skill progress plateaus—participants may reduce their attendance even if the social environment is supportive. The validation of Hypothesis H7 illustrates a complete motivational mechanism: intrinsic motivation strengthens sport commitment, which in turn leads to sustained behavioral intention. This process operates independently of community belonging, echoing real-life observations where participants continue not because they are emotionally bound to a group, but because they value the personal transformation that the sport itself brings.

### 5.4 Practical implications

The findings of this study provide valuable insights for the localization and sustainable development of CrossFit in China.

First, prior evidence demonstrates that intrinsic motivation enhances exercise commitment through health, competence, and enjoyment, thereby fostering continuous participation intentions. This mediating pathway is critical for retaining Chinese CrossFit enthusiasts. Accordingly, operators should develop strategies centered on activating core motivations and consolidating exercise commitment, which may be implemented in three ways: Strengthening health motivation by establishing a perceptible and trackable health benefit system. Given that health motivation is the strongest predictor of sustained participation—and that Chinese participants value tangible outcomes—gyms should convert abstract health concepts into measurable experiences. For example, personalized health records could reinforce the perception that “exercise = health benefits.” Activating competence motivation through a low-barrier, stepwise skill development system. As CrossFit’s high technical threshold and delayed gratification may weaken competence motivation, strategies should lower entry barriers and provide continuous feedback. Introductory movement breakdown courses or novice-friendly WODs can yield early positive experiences, while “skill unlock walls” or “challenge events” can quantify progress for advanced participants. Enhancing enjoyment motivation by integrating incremental achievements with light social interaction. Although weaker than health and competence motivations, enjoyment reduces dropout risk by countering perceptions of monotony or excessive intensity. Initiatives such as Family Training Days or Outdoor Fitness Challenges can foster group encouragement, increase enjoyment, and indirectly reinforce commitment.

Second, results show that community belongingness neither directly predicts continuous participation intention nor serves as a mediator. This result reflects Chinese participants’ prioritization of tangible outcomes (health and competence gains) over emotional group connections. Therefore, operators should reposition community functions as supportive mechanisms for strengthening exercise commitment rather than as retention drivers. For instance, a training-partner matching system based on frequency, goals, and skill level could encourage instrumental socialization, reduce training inertia via peer supervision, and consolidate commitment.

In summary, CrossFit’s localization in China should prioritize inward-oriented commitment by leveraging health, competence, and enjoyment as primary motivators, while employing functionalized communities as supplementary mechanisms. Investment should focus on coaching expertise, equipment upgrades, and health monitoring technologies. By adopting a differentiated strategy that emphasizes tangible outcomes over emotional bonds, operators can reduce costs, mitigate user attrition and gym closures, and ultimately promote the sustainable development of CrossFit in China.

### 5.5 Innovations

Theoretical contributions. This study advances the literature in three key ways. First, it establishes an integrated theoretical model of intrinsic motivation → community belongingness/exercise commitment → continuous participation intention, thereby filling a gap in the CrossFit domain regarding the complete “motivation–mediator–outcome” logic chain. Second, it incorporates belongingness theory and commitment theory into the CrossFit context, extending the applicability of these frameworks to a specialized exercise domain. Third, by conducting empirical analysis with a Chinese sample, it supplements sports participation theory from a cross-cultural perspective and addresses the regional concentration of existing research.

Sample-level contributions. This study is the first to systematically investigate the mechanisms of sustained participation among Chinese CrossFit enthusiasts. Against the backdrop of practical challenges limiting the development of CrossFit in China, the research directly addresses the issue of participant attrition. By analyzing 525 valid responses, this study quantitatively verifies the localized characteristics of Chinese CrossFit participants, thereby filling a gap in research on sustained participation among non-Western populations and providing crucial evidence from a Chinese sample for the cross-cultural extension of sports participation theory.

Practical contributions. This study provides localized empirical evidence for China’s fitness industry and generates actionable recommendations for CrossFit operators. Specifically, it provides guidance on enhancing member retention and refining community engagement strategies, thereby facilitating the effective promotion and sustainable growth of CrossFit in China.

## 6 Research limitations and future research directions

### 6.1 Limitations of the study

Although this study sheds light on the core mechanisms underlying sustained participation among Chinese CrossFit enthusiasts, several limitations should be acknowledged:

First, methodological and design limitations. The use of questionnaires to assess exercise motivation, continuous participation intentions, and related constructs may introduce bias and reduce objectivity. Because questionnaire data rely on structured, predefined items, they may fail to capture the more complex and nuanced inner thoughts of participants. Additionally, the cross-sectional design captures associations between motivation and behavior at a single time point, without considering the long-term dynamics.

Second, sample limitations. Due to practical constraints, only 525 valid responses were obtained, all of which were based on self-reported data. The recruitment process may have introduced selection bias, thereby limiting the objectivity and representativeness of the findings. Furthermore, participants were recruited exclusively through online communities of affiliated gyms, excluding individuals who had already discontinued their participation in CrossFit. However, the “reasons for dropout” among such individuals represent critical comparative information for understanding sustained participation.

Third, variable limitations. This study focused only on the mediating roles of community belongingness and exercise commitment in the relationship between exercise motivation and continuous participation intention. Other potentially important moderating factors—such as perceived training effectiveness (e.g., fitness improvements) and gym service quality (e.g., coach professionalism)—were not included. These factors may influence continuous participation intentions indirectly by strengthening or weakening exercise commitment.

### 6.2 Future research directions

First, expand the sample size and incorporate dropout participants. Future studies should expand the sample pool and specifically recruit individuals who previously participated in CrossFit but have since discontinued their participation. By establishing a comparative framework between “persistent participants” and “dropout participants,” researchers can address the current gap of focusing solely on active users while neglecting those who have discontinued, thereby offering a more comprehensive understanding of sustained participation.

Second, adopting mixed-method approaches. Beyond large-scale questionnaires that ensure statistical representativeness, future research could integrate qualitative methods such as semi-structured interviews. In-depth interviews may help uncover the deeper reasons why CrossFit participants either persist or discontinue, thus providing richer qualitative support for interpreting quantitative relationships.

Third, refining the theoretical model. Future studies should incorporate individual difference variables (e.g., gender, income, family support, and flexibility of working hours) and environmental factors (e.g., perceived training effectiveness and gym service quality) as potential moderators. Analytical approaches such as hierarchical regression or moderation analysis can be employed to explore their roles in the relationships among exercise motivation, exercise commitment, community belongingness, and continuous participation intentions. Such efforts would help develop a more comprehensive theoretical model and offer stronger guidance for practical interventions.

## 7 Conclusion

This study reveals that the continuous participation of Chinese CrossFit participants is primarily driven by the realization of self-value, highlighting the central role of intrinsic motivation over external or community-based factors. These findings offer practical implications for managing fitness communities and designing effective interventions to motivate exercise. Future research should build on these results to further refine the theoretical framework and enhance its practical applicability, thereby contributing to both academic understanding and real-world interventions aimed at promoting sustained engagement in high-intensity training contexts, such as CrossFit.

## Data Availability

The raw data supporting the conclusions of this article will be made available by the authors, without undue reservation.
