# Supplementary material for: An empirical study of continuous participation intention in Chinese CrossFit participants: mediating roles of community belonging and sport commitment
Source: Front Psychol. 2025 Sep 26;16:1674562. doi: 10.3389/fpsyg.2025.1674562 (PMC12511082; doi:10.3389/fpsyg.2025.1674562)
Supplement: Supplementary file 1 [file Table_1.DOCX]

Supplementary Material

# Supplementary Tables

| **Table S1 Exercise motivation scale.** | |
| --- | --- |
| **Serial number** | **Measurement items** |
| EM1 | I engage in CrossFit to cultivate a strong, healthy physique. |
| EM2 | I engage in CrossFit to regulate body weight. |
| EM3 | I engage in CrossFit to participate in stimulating recreational activities. |
| EM4 | I engage in CrossFit to acquire new athletic skills. |
| EM5 | I engage in CrossFit to build new social connections. |
| EM6 | I engage in CrossFit to maintain physical and mental well-being. |
| EM7 | I engage in CrossFit to preserve or enhance my physique. |
| EM8 | I engage in CrossFit to maintain a positive mood. |
| EM9 | I engage in CrossFit to improve existing athletic skills. |
| EM10 | I engage in CrossFit to strengthen social bonds. |
| EM11 | I engage in CrossFit to lead a healthy lifestyle. |
| EM12 | I engage in CrossFit to enhance physical attractiveness. |
| EM13 | I engage in CrossFit to experience a fulfilling life. |
| EM14 | I engage in CrossFit to maintain current athletic proficiency. |
| EM15 | I engage in CrossFit to maintain healthy social relationships. |

| **Table S2. Sense of community belonging scale.** | |
| --- | --- |
| **Serial number** | **Measurement items** |
| CB1 | When I am at the gym (Box), I perceive myself as an integral member of the community. |
| CB2 | I maintain robust interpersonal relationships with the individuals at the gym (Box). |
| CB3 | I perceive that I am accepted by the other members of the gym (Box). |
| CB4 | At the gym (Box), I experience a sense of inclusion. |

| **Table S3 Sport commitment scale.** | |
| --- | --- |
| **Serial number** | **Measurement items** |
| SC1 | I am resolutely determined to continue participating in CrossFit training. |
| SC2 | I am highly engaged in CrossFit training. |
| SC3 | I am committed to persisting with CrossFit training. |
| SC4 | I find it difficult to discontinue CrossFit training. |
| SC5 | I will exert every effort to maintain my participation in CrossFit training. |

| **Table S4 Continuous participation intention scale.** | |
| --- | --- |
| **Serial number** | **Measurement items** |
| CPI1 | In the future, I intend to continue participating in CrossFit training. |
| CPI2 | Even if offered the opportunity to engage in other sports, I will continue participating in CrossFit training. |
| CPI3 | I will recommend CrossFit training to others. |
| CPI4 | I will invite others to join me in CrossFit training |
